# Anti-Inflammatory Effects and Human Skin Safety of the Eastern Traditional Herb *Mosla japonica*

**DOI:** 10.3390/life15030418

**Published:** 2025-03-07

**Authors:** Hyun-Ju Han, Chang-Gu Hyun

**Affiliations:** Jeju Inside Agency and Cosmetic Science Center, Department of Chemistry and Cosmetics, Jeju National University, Jeju 63243, Republic of Korea; 00guswn00@naver.com

**Keywords:** anti-inflammatory effects, COX-2, iNOS, MAPK, IκBα, RAW 264, *Mosla japonica*, skin irritation test

## Abstract

Traditional knowledge has long provided natural solutions for disease prevention and treatment, complementing modern medicine. *Mosla japonica* (Korean mint) has been traditionally valued for its pesticidal, dehumidifying, anti-swelling, and detoxifying properties. This study explores its anti-inflammatory potential using *M. japonica* extract (MJE) in LPS-stimulated RAW 264.7 macrophages and evaluates its safety for human skin applications. MJE significantly reduced inflammatory mediators such as nitric oxide (NO), prostaglandin E_2_ (PGE_2_), and key cytokines (IL-1β, IL-6, TNF-α) in a dose-dependent manner. It also suppressed the expression of iNOS and COX-2, enzymes crucial for inflammation. Mechanistically, MJE inhibited NF-κB activation by stabilizing IκBα, thereby reducing inflammation-related gene expression. Additionally, it downregulated ERK, JNK, and p38 in the MAPK signaling pathway, further contributing to its anti-inflammatory effects. A primary skin irritation test confirmed MJE’s safety, showing no significant skin reactions at 100 μg/mL. These findings highlight MJE’s strong anti-inflammatory properties and potential for dermatological applications. This study underscores the pharmacological value of *M. japonica* and its integration into modern scientific research, aligning with global biodiversity frameworks such as the Nagoya Protocol. Future research may further expand its applications in medicine and skincare.

## 1. Introduction

Traditional medicine in Korea, Japan, and China demonstrates a strong connection between the natural environment and human health by utilizing regionally available medicinal plants to develop advanced medical systems [1,2]. In Korea, the distinct four seasons and abundant herbal resources have contributed to the development of Hanbang, or traditional Korean medicine, which integrates Chinese-origin medicine with indigenous folk remedies. In Japan, traditional medicine, known as Wakanyaku, is rooted in the philosophy of “medicine and food as one”. This concept has been applied across fields such as functional foods, herbal medicine, and traditional cuisine. It emphasizes the idea that food and medicine share the same origin and highlights the importance of food in promoting health and preventing disease by serving a role similar to that of medicine [3,4,5,6,7]. In China, the vast and diverse climatic and environmental conditions have supported the development of traditional medicine and pharmacognosy. Acupuncture is a key aspect of traditional Chinese medicine and has been validated through neurological research for its effectiveness in relieving pain and maintaining bodily balance, offering proven analgesic and circulatory benefits [8,9,10].

In the context of global biodiversity and sustainability, the Nagoya Protocol emphasizes the fair utilization and benefit-sharing of genetic resources and traditional knowledge (TK). This framework highlights the pharmacological potential of medicinal plants, positioning them at the forefront of modern drug development and health-related applications. Moreover, collaborative efforts with local communities ensure the ethical and sustainable use of biodiversity resources [11,12,13].

Among medicinal plants, the *Mosla* genus (Lamiaceae) represents a significant regional botanical resource. *Mosla chinensis* and *Mosla scabra* possess notable pharmacological properties, including antiviral, anti-allergic, and anti-inflammatory effects, primarily due to bioactive compounds such as lignans, flavonoids, and monoterpenoids. These compounds contribute to their therapeutic value in alternative medicine. However, despite this potential, the pharmacological properties of *Mosla japonica* remain largely unexplored, with no existing studies on its anti-inflammatory activity [14,15,16,17,18].

The *Mosla* genus, consisting of fifteen species, is predominantly found in Korea, Japan, and the Ryukyu Islands and has long been used in traditional medicine. *M. chinensis* is commonly employed for treating colds, fever, diarrhea, and digestive disorders, while *M. scabra* is used for respiratory and gastrointestinal diseases, including viral infections, allergies, and gastroenteritis. Due to their diverse pharmacological properties, most research has focused on *M. scabra* and *M. chinensis*, with increasing attention to their chemical composition and biological activities. Given its potential, further investigation is essential to uncover the therapeutic applications of *M. japonica* and expand the understanding of its bioactive properties [19,20,21].

This study investigated the anti-inflammatory effects of *M. japonica* methanol extract (MJE) on LPS-stimulated RAW 264.7 macrophage cells and analyzed its association with the NF-κB and MAPK signaling pathways, which play critical roles in regulating inflammatory responses by controlling the expression of pro-inflammatory mediators and cytokines [22,23,24]. Furthermore, the potential of MJE as a cosmetic ingredient was evaluated through human skin patch tests, aiming to explore its potential for skin health and pharmacological applications. The findings highlight the pharmacological potential of *M. japonica*, emphasizing the integration of traditional knowledge with modern scientific approaches under international frameworks like the Nagoya Protocol. This study aims to contribute to the sustainable and ethical utilization of biodiversity while fostering the development of innovative applications rooted in traditional medicinal resources.

## 2. Materials and Methods

### 2.1. Materials and Reagents

The methanol extract of *M. japonica*, prepared from its whole parts, was purchased from the Natural Product Central Bank, Korea Research Institute of Bioscience and Biotechnology (Cheongju-si, Chungcheongbuk-do, Republic of Korea; sample number: KPM013-045). Gallic acid, quercetin, Folin–Ciocalteu reagent, lipopolysaccharide (LPS), Griess reagent, NS-398, and N_6_-(1-iminoethyl)-L-lysine (L-NIL) were obtained from Sigma-Aldrich (St. Louis, MO, USA). Dulbecco’s Modified Eagle’s Medium (DMEM), fetal bovine serum (FBS), and penicillin/streptomycin (P/S) were purchased from Gibco (Grand Island, NY, USA), and the BCA protein assay kit was purchased from Thermo Fisher Scientific (Waltham, MA, USA). The anti-iNOS antibody was acquired from Merck Millipore (Burlington, MA, USA), and the anti-COX-2 antibody was obtained from BD Biosciences (Franklin Lakes, CA, USA). Primary antibodies, including P-ERK, P-JNK, P-p38, P-IκB-α, T-ERK, T-JNK, T-p38, T-IκB-α, and β-actin, as well as secondary antibodies and a protease/phosphatase inhibitor cocktail, were purchased from Cell Signaling Technology (Beverly, MA, USA). The MAPK inhibitors SB203580 (sc-3533, p38 MAPK inhibitor), SP600125 (sc-200635, JNK inhibitor), and PD98059 (sc-3532, MEK1/2 inhibitor targeting MAPK/ERK pathway) were obtained from Santa Cruz Biotechnology (Dallas, TX, USA). Phosphate-buffered saline (PBS), MTT, dimethyl sulfoxide (DMSO), RIPA buffer, Tris-buffered saline (TBS), and enhanced chemiluminescence (ECL) kits were purchased from Biosesang (Seongnam, Gyeonggi-do, Republic of Korea).

### 2.2. Measurement of Total Phenolic and Total Flavonoid Content

The total phenolic content (TPC) of MJE was measured using the Folin–Ciocalteu method, which is based on the reduction of Folin–Ciocalteu reagent by phenolic compounds, leading to the formation of a molybdenum blue complex [22]. A 2 mg sample was mixed with 1 mL of 80% ethanol and subjected to shaking extraction at 30 °C for 60 min. The extract was then centrifuged for 10 min, and the supernatant was collected for analysis. For the assay, 100 µL of the sample was mixed with 100 µL of 2% Na_2_Cl_3_, followed by 100 µL of 50% Folin–Ciocalteu reagent, and vortexed for 30 s. The reaction mixture was incubated in the dark for 30 min, and the absorbance was measured at 750 nm. Gallic acid was used as the standard to construct the calibration curve, and the TPC was expressed as mg gallic acid equivalent (GAE) per g dry weight. The standard calibration curve for gallic acid had an R² value of 0.9998. The total flavonoid content (TFC) was determined based on a modified method of Jia et al. [23]. A 1 mL aliquot of the extract was mixed with 30 µL of 5% NaNO_2_ and allowed to react at room temperature for 5 min. Subsequently, 30 µL of 10% AlCl_3_ and 200 µL of 1 M NaOH were added to the mixture. The absorbance was measured at 510 nm. Quercetin was used as the standard to construct the calibration curve, and the TFC was expressed as mg quercetin equivalent (QE) per g dry weight. The standard calibration curve for quercetin had an R² value of 1.

### 2.3. Cell Culture

RAW 264.7 macrophage cells were obtained from the Korean Cell Line Bank (KCLB NO. 40071). The cells were cultured in DMEM supplemented with 10% FBS and 1% P/S at 37 °C in a 5% CO_2_ incubator. The medium was replaced every two days.

### 2.4. Cell Viability Assay

Cell viability was assessed through the MTT assay. RAW 264.7 cells (1.5 × 10⁵ cells per well) were plated in 24-well plates and incubated with MJE and LPS (1 μg/mL) for 24 h. The culture medium was replaced with 0.2 mg/mL MTT (500 μL), and the cells were incubated at 37 °C for 3 h. The medium was then removed, and the formazan product was dissolved in DMSO. Absorbance was measured at 570 nm using a microplate reader (Biotek, Winooski, VT, USA).

### 2.5. Measurement of Nitric Oxide (NO) Production

NO levels in the culture supernatant were quantified as nitrite concentrations using Griess reagent. RAW 264.7 cells (1.5 × 10⁵ cells per well) were cultured in 24-well plates with MJE and LPS (1 μg/mL) for 24 h. L-NIL (40 μM), a selective iNOS inhibitor, served as a positive control. Supernatants (100 μL) were combined with an equal volume of Griess reagent in a 96-well plate and incubated at room temperature for 10 min. The absorbance was recorded at 540 nm using a Biotek microplate reader (Winooski, VT, USA).

### 2.6. Measurement of Prostaglandin E (PGE)_2_ and Pro-Inflammatory Cytokines

The levels of PGE_2_, interleukin-6 (IL-6), IL-1β, and tumor necrosis factor-α (TNF-α) in culture supernatants were quantified using ELISA kits following the manufacturer’s instructions. RAW 264.7 cells (1.5 × 10⁵ cells per well) were incubated in 24-well plates with MJE and LPS (1 μg/mL) for 24 h. To assess PGE_2_ levels, NS-398 (100 nM), a selective COX-2 inhibitor, was used as a positive control. Protein concentrations were determined by measuring absorbance at 405 nm or 450 nm using a Biotek microplate reader (Winooski, VT, USA).

### 2.7. Western Blot Analysis

RAW 264.7 cells (6.0 × 10⁵ cells/dish) were seeded in 60 mm dishes and incubated for 24 h. Cells were treated with MJE and LPS (1 μg/mL) for varying time points. After treatment, cells were washed with 1× PBS and lysed in RIPA buffer containing 1% protease inhibitor cocktail at 4 °C for 20 min. Cell lysates were centrifuged at 15,000 rpm for 20 min at −8 °C to separate the supernatant. Protein concentrations were determined using the BCA protein assay kit and standardized to 30 μg/mL. The proteins were then combined with 2× Laemmli sample buffer in a 1:1 ratio and subjected to heat treatment at 100 °C for 5 min. MJE samples were separated by SDS-PAGE and transferred onto a PVDF membrane. Membranes were blocked in 5% skim milk in TBS-T (Tris-buffered saline with 1% Tween 20) for 2 h, incubated overnight at 4 °C with primary antibodies (1:2000 dilution), and then with secondary antibodies (1:1000 dilution) at room temperature for 2 h. Protein bands were detected using an ECL kit and visualized with a Chemidoc imaging system (Vilber Lourmat, Collégien, France).

### 2.8. Primary Skin Irritation Test

This study assessed the potential for primary skin irritation caused by MJE, following the guidelines set by the Ministry of Food and Drug Safety (MFDS) of South Korea, the Personal Care Products Council (PCPC), the Standard Operating Procedures (SOPs) of Dermapro Ltd. (Seoul, Republic of Korea), and the Good Clinical Practice (GCP) principles. The study protocol was reviewed and approved by the Institutional Review Board (IRB) of Dermapro Ltd., ensuring both ethical and scientific validity (IRB No.: 1-220777-A-N-01-DICN22252). All participants provided informed consent prior to their inclusion in the study, consistent with the ethical principles outlined in the Declaration of Helsinki. The study enrolled healthy male and female volunteers aged 20–60 years, free from dermatological conditions, who met the inclusion criteria and did not meet the exclusion criteria. Participants were thoroughly informed of the study objectives, procedures, and potential adverse reactions before providing written consent to participate. The evaluation process included a comprehensive review of the participants’ medical history and baseline characteristics to confirm their eligibility. The designated test area (the dorsal region) was cleansed with 70% ethanol prior to the application of the test material. A volume of 20 µL of the extract was applied to the test site using an occlusive patch, which was left in place for 24 h. Skin reactions were assessed twice: first, 20 min after patch removal, and second, 24 h after removal. The evaluation of skin irritation was conducted in accordance with the PCPC guidelines, and mean irritation scores were calculated for each test material. For the purposes of this study, the maximum grade for irritation reactions was capped at +4, as higher grades (+5) are typically indicative of allergic rather than irritant reactions. The skin response at each test stage was determined using the following formula:

$$\mathrm{Response}=\frac{\sum \left(Grade\times No.\;of\;Responders\right)}{4(Maximum\;Grade)\times n(Total\;Subjects)}\times 100\times 1/2$$

### 2.9. Statistical Analysis

All data were presented as the mean ± standard deviation (SD) from three independent experiments. Statistical significance was evaluated using Student’s *t*-test, with significance levels indicated as # *p* < 0.001 vs. the unstimulated control group and * *p* < 0.05, ** *p* < 0.01, *** *p* < 0.001 vs. the LPS-only group.

## 3. Results and Discussion

### 3.1. The Effect of MJE on Cell Viability in RAW 264.7 Cells

RAW 264.7 macrophages are widely utilized as a representative in vitro model system in inflammation research. They are an immortalized cell line derived from the macrophages of Balb/c mice, transformed by *Abelson murine leukemia virus* (Ab-MLV). These cells respond to external stimuli, such as LPS, by producing various inflammatory cytokines, including TNF-α and IL-6, as well as inflammatory mediators such as NO and PGE_2_. Thus, RAW 264.7 cells are considered highly useful tools for elucidating the molecular mechanisms of inflammation and evaluating the efficacy of potential anti-inflammatory compounds [24,25]. Moreover, RAW 264.7 cells retain key macrophage functions, including phagocytosis and inflammatory mediator production, while being easy to culture, sensitive to experimental conditions, and highly reproducible. Consequently, they serve as an ideal model for immune- and inflammation-related studies. Based on these advantages, this study utilized RAW 264.7 cells to investigate the anti-inflammatory effects of MJE. Meanwhile, cytotoxicity assessment is an essential initial step in evaluating potential anti-inflammatory compounds. This is because cytotoxic substances can cause cell membrane damage or abnormal release of inflammatory mediators, potentially distorting the evaluation of anti-inflammatory effects. Therefore, determining the appropriate experimental concentrations and the maximum permissible concentration in macrophages is crucial [26,27]. Cytotoxicity assessment ensures the safety, efficacy, and validity of experimental results.

**Figure 1:**
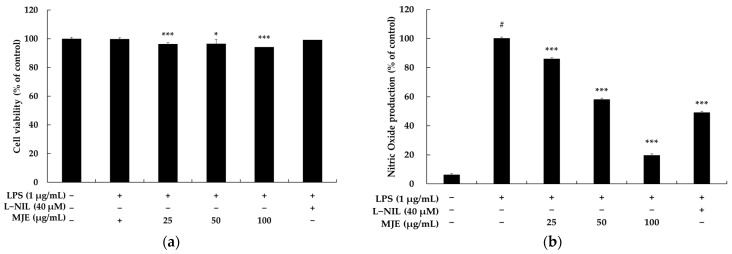
The effect of *Mosla japonica* extract (MJE) on cell viability (**a**) and NO production (**b**) in lipopolysaccharide (LPS)-stimulated RAW 264.7 macrophages. Cells were treated with MJE (25, 50, and 100 μg/mL) and LPS (1 μg/mL) for 24 h, with N6-(1-iminoethyl)-L-lysine (L-NIL, 40 μM) as a positive control. Cell viability is expressed as a percentage relative to untreated control cells, and NO production was quantified. Statistical significance is indicated as # *p* < 0.001 vs. the unstimulated control group and * *p* < 0.05, *** *p* < 0.001 vs. the LPS-only group.

In this context, as an initial step, the cytotoxicity of MJE on RAW 264.7 cells was evaluated using the MTT assay. As is widely recognized, the MTT assay is a reliable method for assessing cell viability by measuring the ability of mitochondria in living cells to reduce the yellow, water-soluble compound MTT into insoluble purple formazan crystals [28,29,30,31]. As shown in Figure 1a, treatment with MJE (25, 50, 100 μg/mL) for 24 h in the presence of LPS (1 μg/mL) revealed no significant cytotoxicity up to a concentration of 100 μg/mL. Additionally, L-NIL (40 μM), a potent and moderately selective inhibitor of inducible nitric oxide synthase (iNOS), used as a positive control, also showed no cytotoxicity. In conclusion, based on these results, subsequent experiments were conducted using concentrations of 25, 50, and 100 μg/mL.

### 3.2. The Effect of MJE on NO Production in RAW 264.7 Cells

NO plays a critical role as a mediator in inflammatory processes and supports various physiological functions, including immune defense and vasodilation, under normal conditions. However, excessive production of NO can exacerbate inflammatory responses and cause tissue damage. Therefore, the ability to inhibit NO production is widely regarded as a key strategy for evaluating the anti-inflammatory potential of compounds [32,33].

Specifically, NO levels in the supernatant of LPS-stimulated RAW 264.7 macrophages are typically measured using the Griess assay. The Griess assay is based on a colorimetric method that quantifies the concentration of nitrite (NO_2_^−^), a stable metabolite of nitric oxide (NOx), in biological samples. In this process, nitrite (NO_2_^−^) reacts with Griess reagents (sulfanilamide and N-(1-naphthyl)ethylenediamine) to form a pink azo compound. The resulting azo compound is stable and has a distinct color, making it suitable for spectrophotometric measurement. Furthermore, the absorbance of this reaction product is detected at 540 nm, allowing for quantitative analysis using a spectrophotometer. Additionally, the Griess assay has the advantage of being sensitive enough to detect even trace amounts of nitrite. However, it cannot measure NO directly and is limited to detecting NO_2_^−^, requiring the conversion of NO to nitrite for analysis. Nonetheless, due to its reliability and simplicity, the Griess assay is widely used in NO production studies and has become an essential tool for investigating NO as an inflammatory mediator [34,35]. As shown in Figure 1b, treatment of RAW 264.7 macrophages with LPS (1 μg/mL) resulted in a more than 10-fold increase in NO production compared to the untreated control, indicating a strong inflammatory response. However, treatment with MJE significantly suppressed LPS-induced NO production in a dose-dependent manner, with inhibition rates of 14.0%, 42.1%, and 80.0% observed at MJE concentrations of 25, 50, and 100 μg/mL, respectively. For comparison, the positive control, L-NIL (40 μM), a well-known iNOS inhibitor, reduced NO production by 51.1%. Notably, the NO suppression effect of L-NIL validates the reliability of the NO inhibition evaluation system used in this study. While L-NIL exhibited slightly higher efficacy than MJE at 50 μg/mL, its inhibitory effect was lower than that observed with MJE at 100 μg/mL. This difference highlights the remarkable effectiveness of MJE, a complex extract, which demonstrates inhibitory potential comparable to or even exceeding that of L-NIL, a pharmacologically established single compound. In conclusion, these findings strongly suggest that MJE possesses significant anti-inflammatory potential and can serve as a promising natural alternative for mitigating inflammatory responses by effectively suppressing NO production.

### 3.3. The Effect of MJE on PGE_2_ and Pro-Inflammatory Cytokine Production in RAW 264.7 Cells

Chronic inflammation is a complex biological process associated with tissue damage, hyperactivation of immune responses, and the secretion of various inflammatory mediators. Notably, PGE_2_ and pro-inflammatory cytokines (IL-1β, IL-6, TNF-α) play pivotal roles in this process. Specifically, PGE_2_, synthesized by the COX-2 enzyme, is a critical inflammatory mediator that contributes to fever, pain, vasodilation, and increased vascular permeability at the site of inflammation. However, excessive production of PGE_2_ in chronic inflammatory conditions leads to tissue damage and promotes the progression of various inflammation-related diseases, including cancer, arthritis, and inflammatory bowel disease (IBD) [36,37,38]. Meanwhile, IL-1β, IL-6, and TNF-α, primarily secreted by immune cells, are crucial in amplifying and regulating inflammatory responses. IL-1β enhances inflammatory signals, contributing to tissue damage and immune cell activation. IL-6 initiates acute inflammatory responses and facilitates the transition to chronic inflammation. In addition, TNF-α acts as a potent pro-inflammatory mediator during the early stages of inflammation, promoting the secretion of other cytokines. Given this, PGE_2_ inhibitors, including COX-2 inhibitors, are effective in alleviating inflammation and pain and are used to relieve symptoms of certain chronic inflammatory diseases. Similarly, cytokine inhibitors (such as TNF-α inhibitors and IL-6R inhibitors) are used to treat autoimmune diseases, including rheumatoid arthritis and Crohn’s disease, with clinically proven efficacy. However, conventional anti-inflammatory drugs, such as NSAIDs and steroids, have limitations, including gastrointestinal disorders and cardiovascular side effects with long-term use. Therefore, developing selective inhibitors targeting PGE_2_ or specific cytokines could offer an effective alternative for controlling inflammation while minimizing side effects. Accordingly, we conducted experiments to evaluate whether MJE could inhibit the production of PGE_2_ and cytokines (IL-1β, IL-6, TNF-α) [39,40].

**Figure 2:**
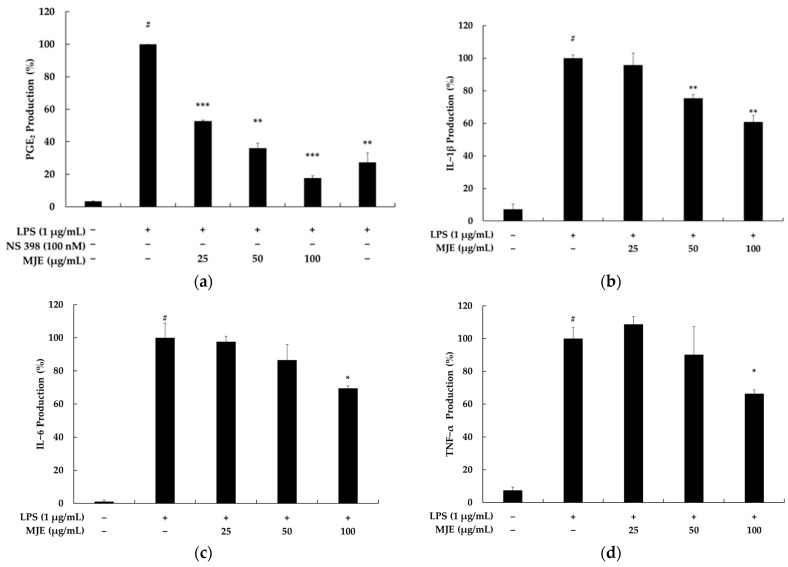
The effect of *Mosla japonica* extract (MJE) on the production of pro-inflammatory cytokines in LPS-induced RAW 264.7 macrophage cells. Cells were treated with MJE (25, 50, and 100 μg/mL) and LPS (1 μg/mL) for 24 h. The levels of (**a**) prostaglandin (PG)E_2_, (**b**) interleukin (IL)-1β, (**c**) IL-6, and (**d**) tumor necrosis factor (TNF)-α were quantified. Statistical significance: # *p* < 0.001 vs. unstimulated control, * *p* < 0.05, ** *p* < 0.01, *** *p* < 0.001 vs. LPS-only group.

In RAW 264.7 macrophages, treatment with LPS (1 μg/mL) significantly increased the production of PGE_2_, IL-1β, IL-6, and TNF-α, with PGE_2_ levels showing a more than 25-fold increase compared to the untreated control. This robust inflammatory response was effectively mitigated by MJE in a dose-dependent manner. Specifically, MJE inhibited PGE_2_ production by 47.2%, 63.9%, and 82.4% at concentrations of 25, 50, and 100 μg/mL, respectively. In comparison, the positive control, NS-398 (a selective COX-2 inhibitor, 100 nM), suppressed PGE_2_ production by 72.7%. While NS-398 showed slightly higher efficacy than MJE at 50 μg/mL, its effect was lower than that of MJE at 100 μg/mL. This indicates that, despite being a complex extract, MJE demonstrates inhibitory effects comparable to those of well-established single-compound pharmacological agents. Furthermore, MJE suppressed the production of pro-inflammatory cytokines in a dose-dependent manner. At concentrations of 25, 50, and 100 μg/mL, MJE reduced IL-1β levels by 4.3%, 24.6%, and 39.1%, respectively (Figure 2). Similarly, it inhibited IL-6 production by 2.4%, 13.4%, and 30.6%. TNF-α levels were also decreased by 9.8% and 33.7% at MJE concentrations of 50 and 100 μg/mL, respectively. These results demonstrate the potent anti-inflammatory properties of MJE, as it effectively suppresses the production of both PGE_2_ and key cytokines (IL-1β, IL-6, TNF-α). The comparable or superior performance of MJE at higher concentrations, relative to the positive control, highlights its potential as a novel and promising natural anti-inflammatory agent. This study suggests that MJE can serve as a viable alternative for mitigating inflammatory responses by targeting multiple inflammatory mediators.

### 3.4. The Effect of MJE on iNOS and COX-2 Protein Expression in RAW 264.7 Cells

iNOS and COX-2 are key enzymes that mediate the production of NO and PGE_2_, respectively, and their expression increases during inflammatory responses, leading to the excessive production of inflammatory mediators. Therefore, the reduction in NO and PGE_2_ concentrations may be attributed not only to the direct inhibition of their biosynthetic pathways but also to other indirect factors, such as the inhibition of enzymatic activity or the depletion of substrates. However, confirming a decrease in iNOS and COX-2 protein expression through Western blot analysis can provide evidence that the tested substance directly suppresses the expression of these proteins, thereby reducing the production of NO and PGE_2_. In conclusion, Western blot analysis plays a critical role in identifying the molecular mechanism of action of the tested substance by verifying changes in the expression of iNOS and COX-2 [41,42,43]. Additionally, it enhances the reliability of the observed inhibitory effects on NO and PGE_2_ production, scientifically supporting the potential of the substance as an inflammation modulator.

As previously mentioned, iNOS and COX-2 play pivotal roles in regulating the production of NO and PGE_2_ during inflammatory responses. Specifically, iNOS is induced by inflammatory stimuli such as LPS and cytokines, leading to the production of NO, while COX-2 metabolizes arachidonic acid to generate prostaglandins, including PGE_2_. These enzymes, by promoting the excessive production of NO and PGE_2_, amplify inflammatory responses and contribute to the pathogenesis of chronic inflammatory diseases.

**Figure 3:**
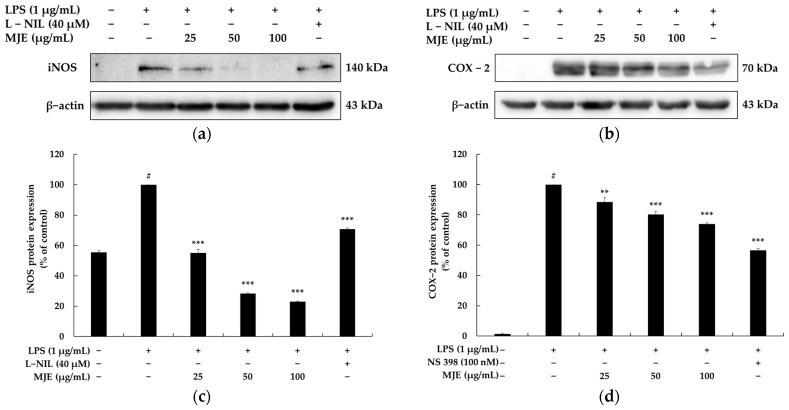
The effect of *Mosla japonica* extract (MJE) on iNOS and COX-2 protein expression in lipopolysaccharide (LPS)-induced RAW 264.7 cells. Cells were treated with MJE (25, 50, and 100 μg/mL) and LPS (1 μg/mL) for 22 h. (**a**,**b**) Western blot analysis of iNOS and COX-2 protein expression. (**c**,**d**) Quantification of iNOS and COX-2 protein levels. N6-(1-iminoethyl)-L-lysine (L-NIL) and NS-398 were used as positive controls for iNOS and COX-2 expression, respectively. Data are presented as mean ± SD from three independent experiments. Statistical significance: # *p* < 0.001 vs. unstimulated control, ** *p* < 0.01, *** *p* < 0.001 vs. LPS-only group.

To elucidate the anti-inflammatory mechanism of MJE, the expression levels of iNOS and COX-2 proteins were evaluated in LPS-stimulated RAW 264.7 macrophages using Western blot analysis. The results revealed that MJE significantly suppressed the expression of both iNOS and COX-2 in a dose-dependent manner at concentrations of 25, 50, and 100 μg/mL. Specifically, MJE reduced iNOS expression by 44.9%, 71.7%, and 76.9% at concentrations of 25, 50, and 100 μg/mL, respectively. Notably, even the lowest concentration of MJE (25 μg/mL) exhibited superior inhibitory efficacy compared to the positive control, L-NIL (40 μM), which reduced iNOS expression by only 29.3%. This highlights the exceptional ability of MJE to suppress iNOS expression, even at relatively low concentrations. In contrast, the suppression of COX-2 expression by MJE was more modest, with inhibition rates of 11.4%, 19.7%, and 26.0% at 25, 50, and 100 μg/mL, respectively. The positive control, NS-398 (40 μM), demonstrated a stronger effect, reducing COX-2 expression by 43.5%. While MJE was less effective than NS-398 in COX-2 inhibition, its ability to significantly suppress iNOS expression, a key mediator of NO production, underscores its potent anti-inflammatory potential (Figure 3). These findings collectively indicate that MJE exerts its anti-inflammatory effects by effectively inhibiting iNOS and, to a lesser extent, COX-2 expression, thereby suppressing the production of inflammatory mediators such as NO and PGE_2_.

### 3.5. The Effect of MJE on the MAPK Signaling Pathway in RAW 264.7 Cells

The MAPK signaling pathway is a key intracellular signaling system that plays a critical role in inflammatory responses and is closely associated with the development of anti-inflammatory agents. Specifically, the MAPK pathway responds to external stimuli such as LPS, cytokines, and stress signals by transmitting signals intracellularly and regulating the expression of inflammatory mediators and cytokines. This pathway, composed of three main components, is involved in various cellular functions, including cell survival, amplification of inflammatory responses, and immune regulation. The major components include ERK, JNK, and p38 MAPK [44,45,46].

Furthermore, the MAPK pathway transmits extracellular signals intracellularly and plays a crucial role in inducing inflammatory responses, being activated by inflammatory stimuli such as LPS. The activation of this pathway is linked to NF-κB activation. Specifically, during the downstream signaling of MAPK, the phosphorylation of ERK, JNK, and p38 promotes the degradation of IκB-α. When IκB-α is degraded, NF-κB is activated and is translocated into the nucleus, where it enhances the transcription of inflammatory genes such as iNOS and COX-2. Therefore, inhibiting the MAPK pathway can block NF-κB activation, thereby reducing the expression of inflammatory genes and effectively suppressing the production of NO, PGE_2_, and pro-inflammatory cytokines (IL-1β, IL-6, TNF-α). As a result, the overactivation of the MAPK pathway contributes to the progression of inflammatory diseases, and targeting this pathway is considered a primary therapeutic goal for alleviating inflammation [47,48,49,50].

In other words, compounds that inhibit the MAPK pathway suppress the production of inflammatory mediators and cytokines, exhibiting potent anti-inflammatory effects. In particular, natural anti-inflammatory substances have been shown to inhibit the MAPK pathway, providing a safe and effective approach to treating inflammatory diseases. For example, tiliroside and 4-methoxyhonokiol have been reported to inhibit the p38 MAPK and JNK pathways, leading to a reduction in COX-2 and iNOS expression [51,52]. Similarly, dihydrofisetin has been shown to effectively suppress NO production and cytokine expression by targeting ERK1/2 and p38 MAPK [53]. In conclusion, the MAPK signaling pathway plays a vital role in regulating the production of inflammatory mediators and cytokines, making it a key target in the development of anti-inflammatory agents. Research targeting MAPK inhibitors offers a promising approach to managing chronic inflammation and inflammatory diseases while laying the foundation for developing natural-product-based anti-inflammatory therapeutics.

**Figure 4:**
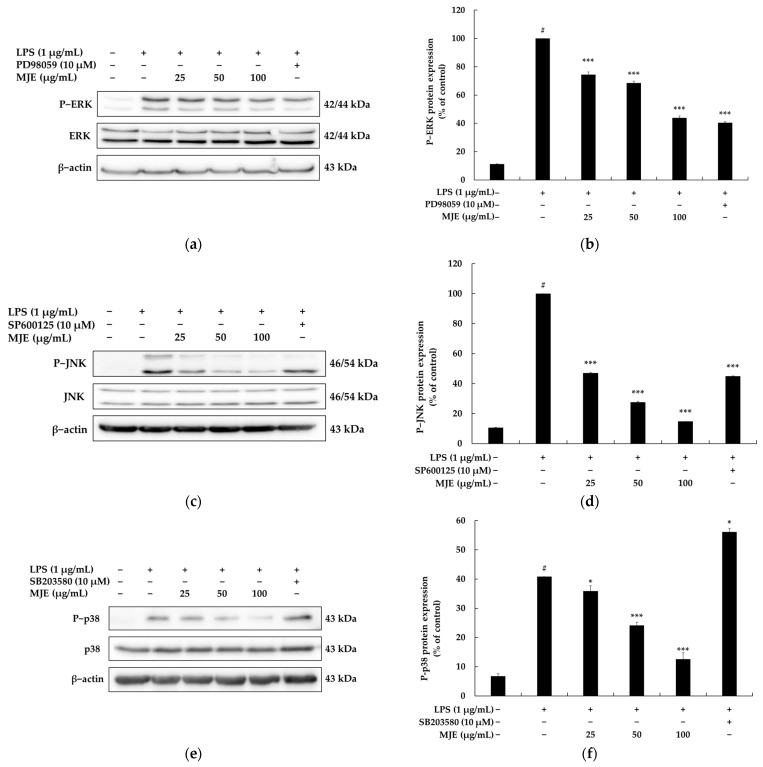
The effect of *Mosla japonica* extract (MJE) on the expression of phosphorylated ERK (P-ERK), JNK (P-JNK), and p38 (P-p38) proteins in lipopolysaccharide (LPS)-induced RAW 264.7 macrophage cells. Cells were treated with MJE (25, 50, and 100 μg/mL) and LPS (1 μg/mL) for 20 min. MAPK inhibitors—PD98059 for ERK, SP600125 for JNK, and SB203580 for p38—were used as controls. (**a**,**c**,**e**) Western blot analysis of P-ERK, P-JNK, and P-p38 protein expression. (**b**,**d**,**f**) Quantification of P-ERK, P-JNK, and P-p38 protein levels. Data are presented as mean ± SD from three independent experiments. Statistical significance: # *p* < 0.001 vs. unstimulated control, * *p* < 0.05, *** *p* < 0.001 vs. LPS-only group.

To investigate the interaction between MJE and the MAPK signaling pathway, the phosphorylation levels of ERK, JNK, and p38 were analyzed in LPS-stimulated RAW 264.7 macrophages. LPS treatment (vehicle + LPS group) significantly increased the phosphorylation levels of ERK, JNK, and p38 compared to the untreated control, indicating the activation of MAPK signaling pathways during the inflammatory response. However, MJE treatment dose-dependently inhibited the phosphorylation of these key signaling molecules, demonstrating its ability to modulate the MAPK pathway. Specifically, MJE suppressed ERK phosphorylation (P-ERK) by 25.6%, 31.5%, and 56.1% at concentrations of 25, 50, and 100 μg/mL, respectively. Similarly, the phosphorylation of JNK (P-JNK) was inhibited by 2.4%, 13.4%, and 30.6% at the same concentrations. For p38 phosphorylation (P-p38), MJE reduced levels by 9.8% and 33.7% at concentrations of 50 and 100 μg/mL, respectively (Figure 4). These results suggest that MJE effectively suppresses LPS-induced inflammatory responses by inhibiting the MAPK pathway, which plays a central role in the production of inflammatory mediators. By reducing the activation of ERK, JNK, and p38, MJE likely contributes to the downstream suppression of iNOS and COX-2 expression, thereby mitigating the production of inflammatory mediators such as NO and PGE_2_. Collectively, these findings highlight the potential of MJE as a natural anti-inflammatory agent targeting the MAPK signaling pathway.

### 3.6. The Effect of MJE on the NF-κB Signaling Pathway in RAW 264.7 Cells

NF-κB is a major transcription factor involved in inflammation, immune responses, cell survival, and differentiation, playing a pivotal role in regulating inflammatory reactions. Typically, NF-κB exists in the cytoplasm as a dimer composed of p65 (RelA) and p50 proteins and is inhibited by the Inhibitor of NF-κB (IκB). Among these inhibitors, IκB-α serves as the primary regulatory protein that binds to NF-κB, preventing its translocation into the nucleus. Accordingly, strategies for inhibiting the NF-κB pathway for developing anti-inflammatory agents can be categorized into two main approaches. First, substances that inhibit the degradation of IκB-α or enhance its expression can indirectly suppress NF-κB activation. Second, directly inhibiting the phosphorylation of p65 or blocking its nuclear translocation can effectively suppress downstream responses of the NF-κB pathway [49,50,54,55]. For instance, metoprolol disrupts the inflammatory response in human cardiomyocytes via β-arrestin2-biased agonism and NF-κB signaling modulation, while carbenoxolone ameliorates allergic airway inflammation through the NF-κB/NLRP3 pathway in mice, and tetramethylpyrazine inhibits the inflammatory response after spinal cord injury by downregulating the TNFR1/IκB-α/NF-κB p65 pathway [56,57,58].

**Figure 5:**
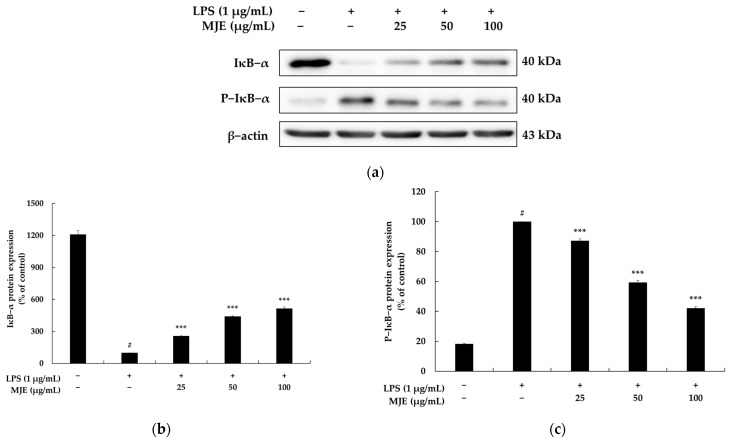
The effect of *Mosla japonica* extract (MJE) on the expression of IκB-α and phosphorylated IκB-α (P-IκB-α) proteins in lipopolysaccharide (LPS)-induced RAW 264.7 macrophage cells. Cells were treated with MJE (25, 50, and 100 μg/mL) and LPS (1 μg/mL) for 20 min. (**a**) Western blot analysis of IκB-α and P-IκB-α protein expression. (**b**,**c**) Quantification of IκB-α and P-IκB-α protein levels. Data are presented as mean ± SD from three independent experiments. Statistical significance: # *p* < 0.001 vs. unstimulated control, *** *p* < 0.001 vs. LPS-only group.

This study focused on the first strategy to suppress NF-κB activation by inhibiting the degradation of IκB-α. To investigate the relationship between MJE and the NF-κB signaling pathway, we evaluated the levels of IκB-α protein expression and the inhibition of IκB-α phosphorylation in LPS-stimulated RAW 264.7 macrophages. In the vehicle + LPS group, LPS treatment significantly increased the phosphorylation of IκB-α (P-IκB-α), indicating enhanced NF-κB activation. However, treatment with MJE dose-dependently suppressed the phosphorylation of IκB-α by 12.8%, 40.8%, and 57.8% at concentrations of 25, 50, and 100 μg/mL, respectively. Conversely, MJE treatment increased IκB-α protein expression levels by 55.8%, 241.8%, and 315.7% at the same concentrations, effectively stabilizing IκB-α and preventing its degradation (Figure 5). These findings suggest that MJE mitigates LPS-induced inflammatory responses by maintaining IκB-α stability and reducing its phosphorylation, thereby inhibiting NF-κB activation. Moreover, as NF-κB activation is closely linked to MAPK signaling pathways, the ability of MJE to suppress key MAPK components such as ERK, JNK, and p38 further supports its regulatory effect on NF-κB activity. In conclusion, this study demonstrates that MJE exerts its anti-inflammatory effects by regulating the NF-κB pathway through the dual mechanism of stabilizing IκB-α and inhibiting its phosphorylation. These results provide strong evidence for the potential of MJE as an effective natural anti-inflammatory agent capable of targeting multiple inflammatory pathways.

### 3.7. Total Phenolic and Flavonoid Content of MJE

The physiological activity of natural products is influenced by various secondary metabolites, with phenolic and flavonoid compounds playing crucial roles in antioxidant and anti-inflammatory functions. These compounds serve as key indicators for the standardization of natural products and are essential for ensuring consistent quality and reproducibility in both research and industry [59,60]. The composition of natural product extracts can vary significantly depending on harvesting time, geographical origin, and extraction methods. Therefore, the quantitative analysis of total phenolic and total flavonoid content is a critical step in maintaining quality uniformity and providing scientific evidence for the efficacy and safety of natural-product-based materials [61,62]. In this study, the total phenolic and flavonoid contents of MJE were measured. The results showed that MJE contained 62.29 ± 3.98 mg GAE/g of total phenolics and 418.01 ± 14.75 mg QE/g of total flavonoids, indicating that MJE is rich in bioactive phenolic and flavonoid compounds. Furthermore, this study highlights the importance of standardization for the industrialization of MJE and is expected to contribute to the establishment of consistent quality control standards for future large-scale production.

### 3.8. Primary Skin Irritation Test of MJE

A primary skin irritation test in humans is a crucial step in evaluating the safety of functional ingredients during the development process. In particular, this test confirms whether the ingredient causes irritation or adverse reactions to the skin, thereby ensuring consumer trust and providing scientific evidence for commercialization. Furthermore, it provides direct interaction data with human skin, offering more reliable results compared to animal experiments. Additionally, the test adheres to the ethical principles outlined in the Declaration of Helsinki and complies with international safety standards, ensuring the ethical validity of the research [63,64,65,66].

Accordingly, this study conducted a primary skin irritation test on MJE at Dermapro Inc., a specialized clinical testing institute for skin. The test involved 30 female volunteers aged 20 to 60 years with no history of irritation or allergic contact dermatitis, with an average age of 44.57 ± 7.26 years. Moreover, the study was approved by the Institutional Review Board (IRB) of Dermapro (IRB No.: 1-220777-A-N-01-DICN22252). All participants were fully informed about the study’s purpose and procedures and provided written consent before participating in the test. During the test, the skin health of each subject was assessed beforehand, and the test area was cleansed with 70% ethanol. Subsequently, 20 µL of MJE at a concentration of 100 μg/mL was applied using an occlusive patch. The patch remained in place for 24 h, and skin reactions were evaluated twice—20 min and 24 h after patch removal. As a result, no noticeable skin reactions were observed in any of the participants, and MJE was evaluated as a hypoallergenic substance in the primary skin irritation test on humans (Table 1). In conclusion, these findings demonstrate that MJE can be safely applied to the skin even at high concentrations, providing significant scientific evidence to support its potential for commercialization as a functional ingredient.

## 4. Conclusions

This study highlights the potent anti-inflammatory properties of *Mosla japonica* extract (MJE) through its ability to modulate key signaling pathways, such as MAPK and NF-κB, in LPS-stimulated RAW 264.7 macrophages. MJE effectively inhibited the production of inflammatory mediators, including nitric oxide (NO), prostaglandin E_2_ (PGE_2_), and pro-inflammatory cytokines (IL-1β, IL-6, TNF-α), in a dose-dependent manner. Mechanistically, these effects were attributed to the suppression of iNOS and COX-2 expression and the inhibition of ERK, JNK, and p38 MAPK phosphorylation, as well as NF-κB activation.

Moreover, the primary human skin irritation test demonstrated the safety of MJE as a topical ingredient, even at high concentrations (100 μg/mL), reinforcing its potential for cosmetic and dermatological applications. These findings emphasize that MJE offers both efficacy and safety, making it a promising candidate for the development of anti-inflammatory therapeutics and functional skincare products.

Importantly, this research underscores the value of integrating traditional knowledge with modern scientific methods under international frameworks like the Nagoya Protocol, which supports the sustainable and ethical utilization of biodiversity. Future studies should further explore the pharmacological potential of MJE, including its effects in vivo and its application in broader medical and cosmetic contexts. Furthermore, identifying the specific bioactive components that are responsible for the anti-inflammatory effects of MJE could provide a deeper understanding of its mechanisms of action and aid in the development of new therapeutics based on natural products.

## Figures and Tables

**Table 1:** Results of human skin primary irritation test (*n* = 30).

No.	Samples	Responders	1st Assessment				2nd Assessment				Reaction Grade (R)
			+1	+2	+3	+4	+1	+2	+3	+4	
1	MJE (100 μg/mL)	0	0	0	0	0	0	0	0	0	0
2	Squalene (Control)	0	0	0	0	0	0	0	0	0	0

MJE refers to the extract of *M. japonica* extract, with squalene used as the positive control. The low irritation category (none to slight) is defined as 0.00 ≤ R < 0.87.

## Data Availability

All data generated or analyzed during this study are fully available within this published article.
